# Estimated Dietary Bisphenol-A Exposure and Adiposity in Samoan Mothers and Children

**DOI:** 10.3390/toxics8030067

**Published:** 2020-09-02

**Authors:** Lacey W. Heinsberg, Christina N.N. Bui, Jennifer C. Hartle, Susan M. Sereika, Courtney C. Choy, Dongqing Wang, Christina Soti-Ulberg, Take Naseri, Muagututia Sefuiva Reupena, Rachel L. Duckham, Jennifer J. Park, Nicola L. Hawley, Nicole C. Deziel

**Affiliations:** 1 Department of Human Genetics, Graduate School of Public Health, University of Pittsburgh, Pittsburgh, PA 15261, USA; 2 Department of Environmental Health Sciences, Yale School of Public Health, New Haven, CT 06510, USA; christina.nn.bui@gmail.com (C.N.N.B.); nicole.deziel@yale.edu (N.C.D.); 3 Department of Public Health and Recreation, College of Health and Human Sciences, San José State University, San José, CA 95192, USA; jennifer.hartle@sjsu.edu; 4 Department of Health and Community Systems, School of Nursing, University of Pittsburgh, Pittsburgh, PA 15261, USA; ssereika@pitt.edu; 5 Department of Epidemiology, International Health Institute, School of Public Health, Brown University, Providence, RI 02912, USA; courtney_choy@brown.edu; 6 Department of Global Health and Population, Harvard T.H. Chan School of Public Health, Harvard University, Boston, MA 02120, USA; dqwang@hsph.harvard.edu; 7 Ministry of Health, Apia, Samoa; ChristinaU@health.gov.ws (C.S.-U.); TakeN@health.gov.ws (T.N.); 8 Lutia I Puava Ae Mapu I Fagalele, Apia, Samoa; smuagututia51@gmail.com; 9 Institute for Physical Activity and Nutrition, Deakin University, Burwood Victoria 3125, Australia; r.duckham@deakin.edu.au; 10 Australian Institute for Musculoskeletal Science, The University of Melbourne and Western Health, St. Albans, Victoria 3021, Australia; 11 Department of Chronic Disease Epidemiology, Yale School of Public Health, New Haven, CT 06510, USA; park.jennifer@aya.yale.edu (J.J.P.); nicola.hawley@yale.edu (N.L.H.)

**Keywords:** exposure assessment, BPA, obesity, environmental disparities

## Abstract

The Pacific Island nation of Samoa is marked by prevalent obesity and an increasing dependence on packaged foods likely to contain the endocrine disruptor bisphenol-A (BPA). We evaluated participant- and household-level characteristics associated with estimated dietary BPA exposure in Samoan mothers and their children and examined associations between dietary BPA exposure and body mass index (BMI) and abdominal circumference (AC). Dietary BPA exposure indices were estimated for 399 mother–child pairs by combining information from dietary questionnaires and relative concentrations of BPA measured in foods/beverages. We observed moderate to strong correlation between mother–child daily BPA indices (Spearman’s rho = 0.7, *p* < 0.0001). In mothers, we observed lower daily BPA indices in those who were less physically active (*p* = 0.0004) and living in homes with higher income (*p* = 0.00001). In children, we observed lower daily BPA indices in those living in homes with higher income (*p* = 0.0003) and following a less modern dietary pattern (*p* = 0.002), and higher daily BPA indices in those who were less physically active (*p* = 0.02). No significant associations were observed between daily BPA indices and BMI or AC. Despite this, the application of the daily BPA index identified factors associated with dietary BPA exposure and warrants further examination in Samoa and other understudied populations.

## 1. Introduction

The prevalence of obesity has almost tripled globally since 1975 and is continuing to rise [1]. Obesity across the lifespan has been linked to many chronic health conditions, including diabetes, osteoarthritis, dyslipidemia, cardiovascular disease, and certain cancers [2]. Although obesity is commonly attributed to high caloric intake, genetic predisposition, and sedentary lifestyles, mounting evidence suggests that environmental exposures to synthetic chemicals in consumer products may also contribute to obesity trends [3,4]. Specifically, increased exposure to certain endocrine-disrupting compounds, which have been termed “obesogens”, may promote or cause obesity by disrupting lipid metabolism or altering hormone levels [3,4,5].

One such chemical that has received a notable amount of attention as a potential obesogen is bisphenol-A (BPA) [6]. BPA is a synthetic monomer used in epoxy resins and polycarbonate plastics as a protective coating to prevent contamination or extend product shelf life and is found in a variety of consumer products including plastic food packaging, reusable food containers, canned goods, bottle tops, baby bottles, dinnerware plastics, and water supply pipes [6,7]. Exposure to BPA is widespread, with detectable levels of BPA in 89.7% of urine samples collected in the United States (U.S.) between 2011 and 2012 [8]. The chemical structure of BPA is remarkably similar to synthetic estrogen and BPA can activate estrogen receptors and interfere with endocrine signaling even at low doses [9]. This estrogen-like activity interferes with the production, release, transport, and metabolism of natural hormones, alters energy balance and fat gain, and is associated with reproductive and developmental toxicity [3,7,10]. Despite this evidence, BPA has not been widely replaced in many food packaging sources and other consumer products, and worldwide production and consumption of BPA have increased over the past decade [11].

The nation of Samoa has among the highest prevalence of obesity in the world with about 65% of the population having the condition [12,13]. Obesity trends have been largely attributed to changing dietary patterns, less active jobs, increasing costs of local staple food production, and more prevalent consumption of inexpensive, imported, micronutrient-poor, and energy-dense convenience foods [12,14]. Notably, these food products include a variety of processed canned foods, canned beverages, and foods wrapped in plastic packaging, many of which are low in nutritional content and are more likely to contain BPA [15,16].

Investigating the role of dietary BPA exposure may be important in identifying modifiable targets for intervention to reduce the burden of obesity and associated long-term health outcomes in Samoa and other vulnerable, low- and middle-income countries and communities. However, BPA exposure assessment for the study of chronic diseases is challenging. Specifically, biological monitoring, typically assessed with quantitative measurements of BPA concentrations in urine, reflects only recent exposure because BPA is rapidly metabolized and excreted with a half-life <7 h [17]. Consequently, urinary BPA measurements collected from the same individual exhibit poor repeatability (among both adults and children), and therefore many samples collected over time are needed to accurately quantify usual exposure [17,18,19]. Furthermore, BPA concentrations in blood samples, which reflect a longer window of exposure, are often low and difficult to detect [20]. Since diet is the dominant source of BPA exposure [21], a survey-based, self-report measure discriminating between high and low dietary exposure could be very useful, particularly for middle- and low-income countries and communities, because of issues related to affordability and access to laboratory testing. Therefore, the purpose of this study was to use a semi-quantitative, survey-based tool, designed for use in Samoa, to identify participant characteristics that are linked to higher estimated dietary BPA exposure and examine the relationship between BPA exposure and measures of adiposity in Samoan mothers and their children.

## 2. Materials and Methods

### 2.1. Study Design, Setting and Sample

This was a cross-sectional, observational analysis of data from the 2017 wave of the ongoing *Ola Tuputupua’e* (“Growing Up”) child cohort study, whose initial design and recruitment procedures have been described elsewhere [22,23]. Briefly, participants were surveyed between June and August 2017 from 11 villages in three regions of the island of Upolu: the Apia Urban Area (urban), Northwest Upolu (peri-urban), and rest of Upolu (rural). Data collection consisted of anthropometric measurements, a dietary questionnaire, and surveys regarding child and maternal health, physical activity, and household characteristics administered by Samoan research assistants. Mothers and children were included in this study if both the mother and child were of Samoan origin based on maternal report of four Samoan grandparents, and children were three to seven years old. No other exclusion criteria were applied. A total of 412 mother–child pairs took part. Maternal informed consent and child assent were obtained, and protocols were approved by the Yale Institutional Review Board (identification code: HIC2000020519; date of approval 1 May 2017) and the Samoa Ministry of Health’s Health Research Committee (date of approval: 27 April 2017).

### 2.2. BPA-Relevant Dietary Data

Dietary data were collected using a 115-item food frequency questionnaire designed to capture Samoa-specific dietary patterns [23]. For the purpose of this study, an additional nine BPA-relevant questions were developed based on a review of the literature and firsthand knowledge of Samoan dietary patterns. Participants were asked how often in the last month they had consumed specific food items in standard serving sizes. An overview of the nine BPA-relevant items [15] from the questionnaire (e.g., *In the past month, how often did you eat foods that were stored in plastic film/packaging while hot (e.g., heated takeaway pies)*) is presented below, with an expanded version available in the Supplementary Material (S1 Methods).

### 2.3. Estimated Dietary BPA Exposure

We applied a semi-quantitative indicator of estimated dietary BPA exposure by assigning each of the 9 food/beverage items queried a relative score ranging from 0 (no/low BPA concentration) to 3 (high BPA concentration). Scores were based on information reported by Tse et al. (2017) and were calculated as part of a systematic review of published literature and expert assessments [24]. In the current study, we used an average of the literature-based and expert-based scores as detailed in the Supplementary Material (Table S1). The scores were then used to weight item-specific daily consumption frequencies reported by participants. This method results in a daily BPA index that aggregates multiple dietary sources of BPA as shown in Equation (1)

(1) $$BPA_{daily}=C_{1}*F_{1}+C_{2}*F_{2}+\cdots C_{9}*F_{9}$$

where $BPA_{daily}$ is the total daily BPA intake index for a given individual; C is a unitless exposure score assigned to a given food/beverage item; and F is the daily consumption frequency for a given individual and given food/beverage item in servings per day [24,25]. The daily BPA index represents a surrogate measure of typical, daily, dietary BPA exposure over the prior month with a higher daily BPA index indicating a higher level of daily BPA exposure. To better understand the impact of relative scoring within our sample, we also used a categorical version of $BPA_{daily}$ by collapsing the continuous variable into tertiles based on daily BPA index distribution.

### 2.4. Participant Characteristic and Household Data

Mothers reported detailed information related to maternal, child, and household characteristics. Household-level characteristics included village of residence, annual household income, and a household asset score, which has been used for several decades in this setting as a proxy for socioeconomic resources [23,26]. Annual household income was categorized into lower (<5000 talā; approximately equivalent to <1900 U.S. dollars), middle (5000 to 9999 talā; approximately equivalent to 1900 to 3799 U.S. dollars), and upper (≥10,000 talā; approximately equivalent to ≥3800 U.S. dollars) income groups. The household asset score is the sum of the number of 18 possible items available from a household inventory list that included a refrigerator, freezer, stereo, portable stereo, microwave oven, rice cooker, blender, sewing machine, television, VCR or DVD player, couch, washing machine, landline telephone, computer or laptop, tablet computer, electric fan, air conditioner, and motorized vehicle [23]. For mothers, age and education were reported to the nearest year and physical activity was estimated using the Global Physical Activity Questionnaire by calculating the total number of self-reported daily moderate to vigorous physical activity (MVPA) minutes [27]. Given an extreme floor effect observed in the MVPA data [28], physical activity was dichotomized as zero MVPA minutes versus greater than zero MVPA minutes.

For children, physical activity levels were estimated using the Netherlands Physical Activity Questionnaire for Young Children in which mothers described their child’s level of physical activity in comparison with other children [29]. Based on the questionnaire structure, data were collapsed into three categories, including maternal reports of children who were less active than their peers, about equal with their peers, and more active than their peers. We used data from the food frequency questionnaire to compute a continuous factor score of dietary patterns for children; the procedures for score creation have been described elsewhere [22,23]. A dichotomized variable was created using the sample median of the modern pattern factor score to separate children into “more modern” or “less modern” dietary pattern groups, with a “more modern” pattern reflecting greater consumption of “westernized foods” such as red meat, condiments, and processed snacks.

### 2.5. Measures of Adiposity

Adiposity measures included body mass index (BMI) and abdominal circumference (AC). Participants were measured while wearing lightweight clothing. Height was measured to the nearest tenth in centimeters (cm) using a stadiometer (Pfister Imports, New York, NY, USA) and weight was measured to the nearest tenth in kilograms (kg) using a digital scale (Tanita Corporation of America, Arlington Heights, IL, USA). Abdominal circumference was measured to the nearest tenth in cm at the level of the umbilicus, rather than waist circumference, since finding the natural waist can be challenging in those with significant abdominal adiposity. All measures were taken in duplicate and averaged for use in analyses.

### 2.6. Statistical Analyses

All statistical analyses were conducted using R version 4.0.1 [30]. Detailed data screening and descriptive analyses were performed. Daily consumption patterns for BPA-relevant food/beverage items as well as item-specific and total daily BPA indices for mothers and children were characterized using medians and interquartile ranges (IQR) and compared using sina with violin plots. The association between the daily BPA indices for mothers and their children was illustrated using scatterplots and summarized using Spearman correlations.

To evaluate associations between participant and household characteristics and daily BPA indices in mothers and children, we conducted multiple linear regression analyses. To stabilize regression parameters and improve model fit, we natural log-transformed the daily BPA index as our outcome variable. Models included all potential exposure determinants identified based on a review of the literature and prior associations with dietary consumption patterns in this population, regardless of statistical significance. Pregnant mothers (*n* = 23) were excluded from these analyses to avoid potential bias related to differing dietary patterns. Data were determined to be missing completely at random and models utilized listwise deletion. Unstandardized regression estimates, 95% confidence intervals (CI), and *p*-values from *t*-test statistics for each model regression coefficient were obtained and model assessment was performed using residual analysis and influence diagnostics. To enhance interpretability of the unstandardized regression coefficients (β) corresponding to a one-unit change in the log-transformed BPA index, we calculated the percent change in daily BPA indices corresponding to each characteristic as shown in Equation (2).

(2) Percent change=[exp(β)−1]∗100

To evaluate the relationships between daily BPA indices and BMI and AC, multiple linear regression models were applied. Variables identified from the literature and those with a *p*-value < 0.05 in preliminary, bivariable analyses were retained in our final model. Pregnant mothers were also excluded from these analyses to avoid bias related to adiposity measures. Our adiposity-related analyses were then repeated using the categorical version of the daily BPA index described above. For all regression models, R^2^ values were reported as the percent of variance in outcomes explained by each model and *p*-values < 0.05 were considered statistically significant.

## 3. Results

### 3.1. Participant Characteristics

Of the 412 mother–child pairs surveyed, 13 pairs were removed due to missing dietary data preventing us from computing BPA indices. Our final overall sample size included 399 mother–child pairs, but sample sizes varied between analyses based on participant characteristic- and outcome-specific data availability. Distributions of demographic and household characteristics were similar between all groups of participants excluded or included in the various analyses.

Participant- and household-level characteristics are presented in Table 1. For mothers, age ranged from 20 to 58 years, with an average (±standard deviation (SD)) of 34.9 (±8.7) years; years of education ranged from 6 to 19 years, with an average of 12.3 (±1.7) years; BMI ranged from 17.4 to 68.9 kg/m^2^, with an average of 34.9 (±6.7) kg/m^2^; AC ranged from 70.5 to 179.3 cm, with an average of 109.3 (±14.6); and physical activity was low, with 73.2% of the sample reporting zero daily MVPA minutes. For children, age ranged from 3.2 to 7.5 years, with an average of 5.3 (±0.9) years; BMI ranged from 12.5 to 30.8 kg/m^2^, with an average of 16.6 (±1.9) kg/m^2^; AC ranged from 43.9 to 90.3 cm, with an average of 55.5 (±5.1) cm; and the sample was 51.4% female. Child physical activity reported by mothers included categories of less active than peers, about equal activity to peers, and more active than peers and accounted for 8.3%, 43.1%, and 48.6% of the overall sample, respectively. For mothers and children, annual household income categories of <5000 talā, 5000 to 9999 talā, and ≥10,000 talā accounted for 59.4%, 23.0%, and 17.6% of our sample, respectively. Household asset scores ranged from 0 to 18, with an average of 5.7 (±4.0) per household.

### 3.2. BPA Indices and Participant Characteristics

Item-specific daily consumption frequencies and BPA indices for mothers and children are presented in Table 2. For mothers, daily BPA indices ranged from 1.71 to 44.02 with a median (IQR) of 11.92 (6.17). Among children, daily BPA indices ranged from 0.52 to 40.70 with a median of 9.97 (5.74) and the distribution was similar between sexes and across categorical ages. Among both mothers and children, item-specific consumption frequencies were similar in magnitude and ordering. The two items with the highest median daily consumption frequency were Item 1 (*Cold beverages from a hard plastic cup*) with a daily median of 3.0 (2.0) servings in mothers and 2.0 (2.0) servings in children and Item 2 (*Hot beverages from a hard plastic cup*) with a daily median of 2.0 (2.0) servings in mothers and 2.0 (2.0) servings in children. Because daily BPA indices were computed as daily consumption frequencies weighted by exposure scores, similar trends were observed in item-specific daily BPA indices as shown in Table 2. However, because Item 2 (*Hot beverages from a hard plastic cup*) was more likely to contain BPA, it yielded the highest contribution to estimated dietary BPA exposure, with a median daily BPA index of 4.0 (4.0) in mothers and children, followed by Item 1 (*Cold beverages from a hard plastic cup*), with a median daily BPA index of 3.99 (2.66) in mothers and 2.66 (2.66) in children.

**Figure 1:**
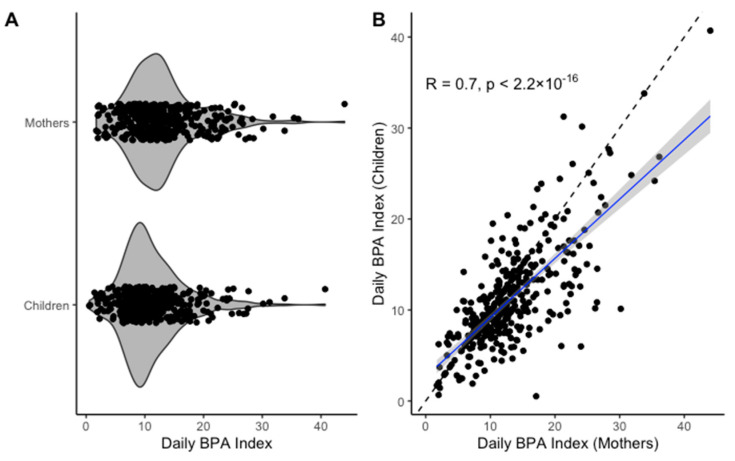
Comparison of daily BPA indices for mothers and children: (**A**) sina with violin plot of daily BPA indices for mothers and children; (**B**) correlation plot of daily BPA indices for mothers and children; R, Spearman’s rho; solid line represents fitted line and dashed line represents *y = x*.

Figure 1 displays the distributions and correlations between daily BPA indices for mothers and children. For the majority of participants, the daily BPA index was between 0 and 20, but there were several values outside of this range as high as 44.0 in mothers and 40.7 in children as shown in Figure 1A. We observed moderate to strong correlation between mother–child daily BPA indices (Spearman’s rho = 0.7, *p* < 0.0001) as shown in Figure 1B. Similar item-specific plots and correlations for mothers and children are presented in the Supplementary Material (Figure S1 and Table S2). Item-specific correlations between mothers and children ranged from 0.5 for Item 2 (*Hot beverages from a hard plastic cup*) to 0.9 for Item 5 (*Food packaged in a metal can*).

The results of multiple linear regression assessing the relationships between participant characteristics and natural log-transformed daily BPA indices for mothers and children are presented in Table 3 and Table 4, respectively. In mothers (Table 3), we observed associations between daily BPA indices and physical activity (*p* = 0.0004) and annual household income (*p* = 0.00001). Specifically, daily BPA indices were 18.9% lower (95% CI = −28.1% to −9.5%) in mothers reporting 0 MVPA minutes compared with mothers reporting >0 MVPA minutes and 28.1% lower (95% CI = −37.5% to −17.3%) in mothers living in homes with an annual household income ≥10,000 talā compared with mothers living in homes with annual household income <5000 talā.

In children (Table 4), we observed associations between daily BPA indices and annual household income (*p* = 0.0003), dietary pattern (*p* = 0.002), and physical activity (*p* = 0.02). Specifically, daily BPA indices were 15.6% lower (95% CI = −24.4% to −5.8%) in children categorized in the “less modern” dietary group compared with the “more modern” dietary group and 25.2% lower (95% CI = −36.2% to −13.1%) in children living in homes with an annual household income ≥10,000 talā compared with mothers living in homes with annual household income <5000 talā. Lastly, daily BPA indices were 20.9% higher (95% CI = 3.1% to 31.0%) in children reported to be less physically active than their peers compared with children reported to be more physically active than their peers. Overall, overlapping results between mothers and children included associations between daily BPA indices and household income and physical activity, though associations with physical activity were in opposite directions for mothers and children.

### 3.3. BPA Indices and Adiposity

In preliminary analyses in mothers, we observed bivariable associations between BMI and AC with age (*p* = 0.01 (BMI) and *p* = 0.01 (AC)), annual household income (*p* = 0.0008 (BMI) and *p* = 0.0004 (AC)), and household asset score (*p* = 0.02 (BMI) and *p =* 0.04 (AC)). Overall, we observed that BMI and AC were higher in older women and those with a higher annual household income and greater number of household assets. In children, we observed associations between BMI and AC with household asset score (*p* = 0.001 (BMI) and *p* = 0.02 (AC)), with higher BMI and AC observed in participants living in homes with more assets. As expected, we also observed associations between AC and age (*p* < 0.0001), with older children having higher AC.

The results of multiple linear regression examining associations between daily BPA index and BMI and AC in mothers and children are presented in Table 5 and Table 6, respectively. Based on existing literature and our preliminary analyses described above, both models included adjustment for age, physical activity, annual household income, and household asset score. In the child-specific analyses, we also adjusted for child sex and dietary pattern group. Because the distribution of the daily BPA index was similar between sexes and across age groups, we chose to control for age and sex rather than stratify in subgroups. In both mothers and children, we observed no association between the daily BPA index and BMI or AC (*p* = 0.96 and *p* = 0.76 in mothers; *p* = 0.42 and *p* = 0.33 in children) while adjusting for covariates and confounders (Table 5 and Table 6). Among mothers, the models explain 6.4% and 6.2% of variation in BMI and AC, respectively; among children, the models explain 7.5% and 17.4% of variation in BMI and AC, respectively. Associations between the daily BPA index as categorical in tertiles and BMI and AC in mothers and children are presented in the Supplementary Material (Tables S3 and S4). Again, no associations were observed between categorical daily BPA index and BMI or AC in mothers or children.

## 4. Discussion

This study was the first to characterize dietary BPA exposure scores and examine associations with measures of adiposity in Samoan mothers and children. This is an important area of investigation because, traditionally, low-income and underserved communities have been disproportionally exposed to environmental toxins [31]. Diet in the Western Pacific, including Samoa, is marked by decreasing food self-sufficiency and increasing dependence on imported, packaged, and processed foods, which have been associated with increased BPA exposure [7,15]. There have been some efforts to phase out BPA in the U.S. [32] as well as in New Zealand [33] and the Philippines [34], which are responsible for a large proportion of imported food in Samoa. However, BPA replacements in consumer products (e.g., bisphenol S and bisphenol F) share structural similarities and endocrine disrupting properties with BPA, and labeling of products as “BPA-free” may be misleading to consumers [32]. Moreover, although BPA has been banned from use in baby bottles and formula containers in several countries [34,35], BPA is still used in many consumer goods, and the increased cost of BPA substitutes have largely isolated the focus of BPA phase-out to organic or more expensive food lines, leaving low-income communities disproportionately vulnerable to related adverse health effects [36].

We identified both participant- and household-level characteristics associated with daily BPA indices. In both mothers and children, we observed lower daily BPA indices in the highest income homes. BPA levels have also been found to be inversely associated with family income in the U.S. [37] and social class in Europe [38]. However, in direct contrast to observations in these higher income countries [39], increased income is associated with greater burden of obesity in Samoa [39]. Historically, during economic progression in low- and middle-income countries, obesity has shifted from being more prevalent in high socioeconomic status groups to low socioeconomic status groups [40]. Despite significant economic growth in Samoa over the last two decades, this shift has not been observed [26]. In other settings, higher income is associated with a greater ability to purchase fresh foods but because there is a high reliance on farming across all income groups in Samoa, the relationships between socioeconomic status, dietary patterns, and health outcomes is more complicated [23,26,41]. It is possible that socioeconomic status impacts adiposity via other pathways in this lower income setting and may be masking any impact of BPA or other environmental chemicals.

Next, in both mothers and children, we observed significant differences in BPA indices based on the level of physical activity, but in opposite directions. Specifically, we observed higher daily BPA indices in more active mothers but less active children. We initially suspected this association might be confounded by income in mothers but observed no difference in physical activity between the low income (<5000 talā) and the high income (≥10,000 talā) groups with 0 MVPA minutes reported in 76.1% and 78.3% of women, respectively. Nevertheless, the association with physical activity and daily BPA index was observed even when controlling for income in our regressions. In children, although the directionality of the association between higher BPA indices and lower physical activity was consistent with our hypothesis, this may be due to a small sample size of participants who were reported by their mothers to be “less active than their peers” (*n* = 33, 8.3%). There is a dearth of research related to BPA exposure and physical activity levels and additional work is needed to better understand this relationship in Samoa.

In children, we observed lower daily BPA indices in those categorized in the “less modern” dietary pattern group, compared to “more modern”. Children following a modern dietary pattern consume higher amounts of “westernized” foods including French fries, condiments, and snacks (i.e., processed, individually packaged items) compared with local foods such as vegetables, fish, and coconut [23]. We did not have dietary pattern data in mothers to compare this finding. However, we observed a moderate to strong correlation between maternal and child BPA indices which is consistent with observations in a biomarker-based study of U.S. mothers and their children [38] suggesting there may be a shared environmental or dietary factor that should be considered. These observations not only offer some corroboration to the BPA index applied here, but also represent a potential opportunity for intervention to reduce BPA exposure in the future (e.g., promoting more local, traditional diets in Samoa).

Despite identifying characteristics associated with dietary BPA exposure, we did not observe associations between dietary BPA indices and adiposity in this sample. In the U.S. and Canada, urinary BPA has been associated with increased odds of BMI-defined obesity in both children and adults even while controlling for covariates such as age, sex, and physical activity [42,43]. However, these observations were in higher income countries and, as noted above, there may be factors that are stronger drivers of adiposity in lower income settings such as Samoa. An additional factor that limits the comparability of this study to existing literature is the extreme adiposity observed in our sample. In mothers specifically, the mean BMI was 34.9 kg/m^2^ with only 19.9% of the sample having a BMI <30 kg/m^2^. This is quite different from comparable studies in higher income countries in which upwards of 75% of the sample had BMI in this range [43]. Despite this, we did identify factors associated with adiposity in mothers and children. Age and socioeconomic status stand out in our results, though annual household income appears to be more important in mothers compared with total number of household assets in children.

While it is possible that dietary BPA exposure may not be as important a driver of adiposity in this sample as other factors, some limitations of this study should be considered in the interpretation of the results. First, detailed health histories of participants were not considered in our analyses. Because this was a community-based sample of mothers and children, participants were included despite a small proportion of mothers reporting diagnoses of hypertension or diabetes; potential associations between BPA exposure and adiposity comorbidities, particularly endocrine disorders, should be explored in the future. Next, the daily BPA index was computed using survey data from a dietary questionnaire and is therefore susceptible to recall bias and subsequent BPA index measurement error. Similarly, our questionnaire only covered the consumption patterns of the prior month; if this duration was not representative of typical dietary patterns, that could also contribute to exposure misclassification. However, the BPA-relevant questions were added to a food frequency questionnaire that was previously developed and validated to estimate long-term intake specifically for the Samoan population [44]. Nevertheless, questionnaires covering a longer duration, or repeated administration of questionnaires over time, may provide more relevant information for obesity-related health outcomes [24].

We relied on published measurements of BPA concentrations and expert raters to assign relative exposure scores, without any measurements of BPA in food or drinks directly consumed by participants. None of the quantitative studies that analyzed foods for BPA concentration and used to develop the exposure scores were conducted in Samoa, though a limited number were performed in New Zealand and China, countries with high imports to Samoa [24]. BPA concentrations can vary across and within food/beverage items and BPA migration can be influenced by many factors [24], [45]. Nonetheless, the index was able to identify contrast in estimated dietary BPA exposure with variability across more than an order of magnitude. Finally, although the primary source of BPA exposure is diet [21], unmeasured sources of BPA exposure not captured by a dietary questionnaire may be important in adverse health outcomes. Future work could include additional exploration and validation of the BPA exposure scores using human biospecimens from the Samoan population to identify potential non-dietary sources of BPA exposure and refine this survey-based tool for future use.

## 5. Conclusions

We observed no significant associations between daily BPA indices and BMI or AC. However, we observed moderate to strong correlation between maternal and child BPA indices and identified socioeconomic and behavioral factors associated with our dietary BPA exposure index, providing information about populations who could benefit from more refined exposure and health studies. In particular, measures of socioeconomic status stood out in our analyses both in relation to the daily BPA index and adiposity measures. We conclude that in Samoa, income may be a driver of adiposity to such an extreme that it may mask an association with BPA. This study lays the foundation for future efforts to examine the complex interplay between BPA exposure, socioeconomic status, physical activity, and nutrition in a population at a high-risk of obesity. In low- and middle-income and understudied communities, simple, affordable, and reliable tools are needed to identify sources of BPA and other environmental exposures to improve long-term health outcomes. The public health utility of the daily BPA index warrants further examination in countries and communities with limited resources to measure BPA directly and repeatedly.

## Figures and Tables

**Table 1:** Participant- and household-level characteristics.

**Participant-Level Characteristics (Mothers)**		
	n	Mean (SD)
Age (years)	399	34.9 (8.7)
Education (years)	399	12.3 (1.7)
BMI (kg/m^2^)	376	34.9 (6.7)
AC (cm)	376	109.3 (14.6)
	n	%
Physical Activity		
0 MVPA Minutes	290	73.2%
>0 MVPA Minutes	106	26.8%
**Participant-Level Characteristics (Children)**		
	n	Mean (SD)
Age (years)	399	5.3 (0.9)
BMI (kg/m^2^)	392	16.6 (1.9)
AC (cm)	392	55.5 (5.1)
	n	%
Physical Activity		
Less than peers	33	8.3%
About equal to peers	172	43.1%
More than peers	194	48.6%
Sex		
Female	205	51.4%
Male	194	48.6%
Dietary Pattern		
Less Modern	199	49.9%
More Modern	200	50.1%
**Household-Level Characteristics**		
	n	%
Region		
Apia Urban Area	131	32.8%
Northwest Upolu	140	35.1%
Rest of Upolu	128	32.1%
Household Income		
<30,000 talā	233	59.4%
30,000 to 90,000 talā	90	23.0%
>90,000 talā	69	17.6%
	n	Mean (SD)
Household Assets (total number)	383	5.7 (4.0)

Overall sample size, *n* = 399; data availability varies as shown.

**Table 2:** Daily consumption patterns and bisphenol-A (BPA) exposure summary.

Food or Beverage Item	Exposure Score ^a^	Mothers (*n* = 399)		Children (*n* = 399)	
		Daily Consumption ^b^, Median (IQR)	Daily BPA Index ^c^, Median (IQR)	Daily Consumption ^b^, Median (IQR)	Daily BPA Index ^c^, Median (IQR)
Item 1: Cold beverages from a hard plastic cup	1.33	3.0 (2.0)	3.99 (2.66)	2.0 (2.0)	2.66 (2.66)
Item 2: Hot beverages from a hard plastic cup	2	2.0 (2.0)	4.0 (4.0)	2.0 (2.0)	4.0 (4.0)
Item 3: Canned beverages	1.67	0.29 (0.29)	0.48 (0.48)	0.29 (0.29)	0.48 (0.48)
Item 4: Disposable, plastic bottled beverages	1	0.14 (0.14)	0.14 (0.14)	0.14 (0.14)	0.14 (0.14)
Item 5: Food packaged in a metal can	3	0.43 (0.43)	1.29 (1.29)	0.43 (0.43)	1.29 (1.29)
Item 6: Unheated/uncooked food packaged in plastic/film wrap	0	0.14 (0.29)	0	0.14 (0.29)	0
Item 7: Hot food stored in plastic film/packaging	0	0.14 (0.29)	0	0.14 (0.14)	0
Item 8: Food heated in the microwave/oven in contact with plastic	2.67	0.14 (0.22)	0.38 (0.58)	0.14 (0.24)	0.38 (0.63)
Item 9: Food microwaved in plastic containers	2.33	0.14 (0.14)	0.33 (0.33)	0.14 (0.14)	0.33 (0.33)
Daily BPA index ^c^			11.92 (6.17)		9.97 (5.74)

BPA, Bisphenol A; IQR, interquartile range; ^a^ exposure score extracted from Tse et al., (2017) [24] supplementary material as an average of literature-based and expert-based exposure scores (Table S1); ^b^ reported dietary intake frequencies as servings per day; ^c^ daily BPA index computed in Equation (1) as a sum of item-specific daily BPA indices presented here.

**Table 3:** Results of multiple linear regression examining associations between participant characteristics and log-transformed (base e) daily BPA index in mothers (*n* = 351).

	Daily BPA Index ^a^				
	β	95% CI ( β)	Percent Change ^b^	95% CI (Percent Change)	*p*
Age (years)	−0.001	−0.01 to 0.01	−0.10	−1.00 to 1.01	0.72
Maternal Education (Years)	−0.01	−0.04 to 0.02	−1.00	−3.92 to 2.02	0.50
Physical Activity					
>0 MVPA Minutes	Reference				
0 MVPA Minutes	−0.21	−0.33 to −0.10	−18.94	−28.11 to −9.52	0.0004
Annual Household Income					
<5000 talā	Reference				
5000 to 9999 talā	0.01	−0.11 to 0.14	1.01	−10.42 to 15.03	0.81
≥10,000 talā	−0.33	−0.47 to −0.19	−28.11	−37.50 to −17.30	0.00001
Household Assets (total number)	0.003	−0.01 to 0.02	0.30	−1.00 to 2.02	0.64
Region					
Apia Urban Area	Reference				
Northwest Upolu	0.003	−0.12 to 0.13	0.30	−11.31 to 13.88	0.97
Rest of Upolu	0.04	−0.08 to 0.17	4.08	−7.69 to 18.53	0.50

BPA, Bisphenol A; ^a^ daily BPA index as computed in Equation (1) and natural log-transformed; β, unstandardized regression coefficient estimate; CI, confidence interval; ^b^ percent change = [exp(β) – 1] × 100; *p*, *p*-value based on the *t*-test for the particular adjusted regression coefficient; MVPA, daily moderate to vigorous physical activity minutes; R^2^, 11.5%; all variance inflation factors < 1.2.

**Table 4:** Results of multiple linear regression examining associations between participant characteristics and log-transformed (base e) daily BPA index in children (*n* = 377).

	Daily BPA Index ^a^				
	β	95% CI ( β)	Percent Change ^b^	95% CI (Percent Change)	*p*
Age (years)	−0.02	−0.08 to 0.03	−1.98	−7.69 to 3.05	0.37
Sex					
Female	Reference				
Male	0.08	−0.03 to 0.18	8.33	−2.96 to 19.72	0.14
Dietary pattern					
More modern	Reference				
Less modern	−0.17	−0.28 to −0.06	−15.63	−24.42 to −5.82	0.002
Maternal Education (years)	−0.02	−0.05 to 0.01	−1.98	−4.88 to 1.01	0.14
Physical Activity					
More than peers	Reference				
About equal to peers	0.15	−0.01 to 0.39	16.18	−1.00 to 47.70	0.07
Less than peers	0.19	0.03 to 0.27	20.92	3.05 to 31.00	0.02
Annual Household Income					
<5000 talā	Reference				
5000 to 9999 talā	0.06	−0.08 to 0.19	6.18	−7.69 to 20.92	0.40
≥10,000 talā	−0.29	−0.45 to −0.14	−25.17	−36.24 to −13.06	0.0003
Household Assets (total number)	−0.01	−0.02 to 0.003	−1.00	−1.98 to 0.30	0.15
Region					
Apia Urban Area	Reference				
Northwest Upolu	−0.07	−0.20 to 0.06	−6.76	−18.13 to 6.18	0.27
Rest of Upolu	−0.04	−0.18 to 0.09	−3.92	−16.47 to 9.42	0.51

BPA, Bisphenol A; ^a^ daily BPA index as computed in Equation (1) and natural log-transformed; β, unstandardized regression coefficient estimate; CI, confidence interval; ^b^ percent change = [exp(β) − 1] × 100; *p*, *p*-value based on the *t*-test for the particular adjusted regression coefficient; MVPA, daily moderate to vigorous physical activity minutes; R^2^, 14.2; all variance inflation factors < 1.2.

**Table 5:** Results of multiple linear regression examining associations between daily BPA index and body mass index and abdominal circumference in mothers (*n* = 346) while adjusting for covariates.

	BMI (kg/m^2^)			AC (cm)		
	β	95% CI	*p*	β	95% CI	*p*
Daily BPA Index ^a^	0.003	−0.12 to 0.12	0.96	−0.041	−0.31 to 0.23	0.76
Age (years)	0.07	−0.01 to 0.16	0.08	0.245	0.07 to 0.42	0.01
Physical Activity						
>0 MVPA minutes	Reference			Reference		
0 MVPA minutes	−0.80	−2.47 to 0.88	0.35	−0.488	−4.09 to 3.11	0.79
Annual Household Income						
<5000 talā	Reference			Reference		
5000 to 9999 talā	−0.27	−1.99 to 1.45	0.76	−1.369	−5.06 to 2.32	0.47
≥10,000 talā	3.27	1.22 to 5.31	0.002	5.756	1.37 to 10.15	0.01
Household Assets	0.12	−0.06 to 0.31	0.20	0.194	−0.21 to 0.60	0.35

BPA, Bisphenol A; BMI, body mass index; AC, abdominal circumference; β, unstandardized regression coefficient estimate; CI, confidence interval; *p*, *p*-value based on the *t*-test for the particular adjusted regression coefficient; ^a^ daily BPA index computed as shown in Equation (1); all variance inflation factors <1.2; R^2^ for BMI model, 6.4%; R^2^ for AC model, 6.2%.

**Table 6:** Results of multiple linear regression examining associations between daily BPA index and body mass index and abdominal circumference in children (*n* = 370) while adjusting for covariates.

	BMI (kg/m^2^)			AC (cm)		
	β	95% CI	*p*	β	95% CI	*p*
Daily BPA Index ^a^	0.02	−0.02 to 0.06	0.42	0.05	−0.05 to 0.15	0.33
Age (years)	0.20	−0.004 to 0.41	0.06	1.90	1.39 to 2.41	1.5 × 10^−10^
Sex						
Female	Reference			Reference		
Male	0.22	−0.17 to 0.62	0.27	0.44	−0.54 to 1.41	0.38
Dietary Pattern						
More modern	Reference			Reference		
Less modern	−0.06	−0.47 to 0.34	0.77	−0.61	−1.62 to 0.40	0.23
Physical Activity						
More active than peers	Reference			Reference		
About the same as peers	−0.22	−0.68 to 0.24	0.35	−0.10	−1.24 to 1.05	0.87
Less active than peers	−0.61	−1.39 to 0.17	0.12	−1.99	−3.92 to −0.06	0.04
Annual Household Income						
<5000 talā	Reference			Reference		
5000 to 9999 talā	−0.48	−0.99 to 0.03	0.07	−0.63	−1.91 to 0.65	0.33
≥10,000 talā	−0.20	−0.80 to 0.41	0.52	0.02	−1.49 to 1.52	0.98
Household Assets	0.11	0.06 to 0.16	0.00004	0.21	0.08 to 0.34	0.002

BPA, Bisphenol A; BMI, body mass index; AC, abdominal circumference; β, unstandardized regression coefficient estimate; CI, confidence interval; *p*, *p*-value based on the t-test for the particular adjusted regression coefficient; ^a^ daily BPA index computed as shown in Equation (1); all variance inflation factors <1.3; R^2^ for BMI model, 7.5%; R^2^ for AC model, 17.4%.

## Supplementary Materials

The following are available online at https://www.mdpi.com/2305-6304/8/3/67/s1, S1 Methods: Full BPA-relevant survey questionnaire; Table S1: BPA-related dietary questions and exposure scores; Figure S1: Sina plots for daily, item-specific BPA indices for mothers and children; Table S2: Spearman’s rank correlations between daily, item-specific BPA indices for mothers and children; Table S3: Results of multiple linear regression examining associations between categorical daily BPA index and body mass index and abdominal circumference in mothers; Table S4: Results of multiple linear regression examining associations between categorical daily BPA index and body mass index and abdominal circumference in children.
