# Supplementary material for: Estimated Dietary Bisphenol-A Exposure and Adiposity in Samoan Mothers and Children
Source: Toxics. 2020 Sep 2;8(3):67. doi: 10.3390/toxics8030067 (PMC7560430; doi:10.3390/toxics8030067)
Supplement: Supplementary file 1 [file toxics-08-00067-s001.pdf]

toxics

Supplementary Material: Estimated Dietary Bisphenol-A Exposure and Adiposity in Samoan Mothers and Children

Lacey W. Heinsberg [1,*,†], Christina N.N. Bui [2,†], Jennifer C. Hartle [3], Susan M. Sereika [4], Courtney C. Choy [5], Dongqing Wang [6], Christina Soti-Ulberg [7], Take Naseri [7], Muagututia Sefuiva Reupena [8], Rachel L. Duckham [9,10], Jennifer J. Park [11], Nicola L. Hawley [11,‡] and Nicole C. Deziel [2,‡]

1. Methods. BPA-relevant survey questions

Matou te fia fesiligia nisi o fesili e faatatau i taumafa ma vaiinu ma auala o loo teuina/fausiaina mai ai.

We would like to ask you some specific questions about food and drinks and the packaging it comes in.

1.1. ITEM 1

a. I le masina ua tuana'i atu, e fa'afia **ona e** inuina ni vaimalulu/vai aisa po'o nisi fagu vai malulu i se ipu uga malo? In the past month, how often did **you** drink COLD water or other cold beverages from a hard plastic cup?
 ____ times per day (taimi i le aso) OR _____ times per week (taimi i le vaiaso)
 OR
 ____ times per month (taimi i le masina) ____ Don't Know (Le iloa)

b. I le masina ua tuana'i atu, e fa'afia ona inuina e **lou alo** ni vai malulu/vai aisa po'o ni fagau vai I se aiu uga malo? In the past month, how often did **your child** drink COLD water or other cold beverages from a hard plastic cup?
 ____ times per day (taimi i le aso) OR _____ times per week (taimi i le vaiaso)
 OR
 ____ times per month (taimi i le masina) ____ Don't Know (Le iloa)

1.2. ITEM 2

a. I le masina ua tuana'i atu, e fa'afia **ona e** taumafaina ni vai vevela, ti, kofe po'o nisi lava vai vela e fa'aaogaina ai le ipu uga malo? In the past month, how often did **you** drink HOT water, tea, coffee, or other hot beverages from a hard plastic cup?
 ____ times per day (taimi i le aso) OR _____ times per week (taimi i le vaiaso)
 OR
 ____ times per month (taimi i le masina) _____ Don't Know (Le iloa)

b. I le masina ua tuana'i atu, e fa'afia ona taumafaina e **lou alo** ni vai vevela, ti, kofe po'o nisi lava vai vela e fa'aaogaina ai le ipu uga malo? In the past month, how often did **your child** drink HOT water, tea, coffee, or other hot beverages from a hard plastic cup?
 ____ times per day (taimi i le aso) OR _____ times per week (taimi i le vaiaso)
 OR
 ____ times per month (taimi i le masina) _____ Don't Know (Le iloa)

1.3. ITEM 3

a. I le masina ua tuana'i atu, e fa'afia **ona e** inuina vaiinu suamalie tu'uapa? (f/t;apainu) In the past month, how often did **you** drink beverages from a can (e.g., soda)?
 ____ times per day (taimi i le aso) OR _____ times per week (taimi i le vaiaso)
 OR

____ times per month (taimi i le masina) _____ Don't Know (Le iloa)

b. I le masina ua tuana'i atu, e fa'afia ona inuina **e lou alo** vaiinu suamalie tu'uapa? (f/t;apainu) In the past month, how often did **your child** drink beverages from a can (e.g., soda)?
____ times per day (taimi i le aso) OR _____ times per week (taimi i le vaiaso)
OR
____ times per month (taimi i le masina) _____ Don't Know (Le iloa)

1.4. ITEM 4

a. I le masina ua tuana'i atu, e fa'afia **ona e** inuina ni vai po'o vaiinu suamalie mai fagu vaiinu uga? (f/t;pepsi/fagu vai) In the past month, how often did **you** drink water or other beverages from a disposable, plastic bottle?
____ times per day (taimi i le aso) OR _____ times per week (taimi i le vaiaso)
OR
____ times per month (taimi i le masina) _____ Don't Know (Le iloa)

b. I le masina ua tuana'i atu, e fa'afia ona inuina **e lou alo** ni vai po'o vaiinu suamalie mai fagu vaiinu uga? (f/t;pepsi/fagu vai) In the past month, how often did **your child** drink water or other beverages from a disposable, plastic bottle?
____ times per day (taimi i le aso) OR _____ times per week (taimi i le vaiaso)
OR
____ times per month (taimi i le masina) _____ Don't Know (Le iloa)

1.5. ITEM 5

a. I le masina ua tuana'i atu, e fa'afia **ona e** taumafa i meaai tu'u apa? (f/t: apa eleni, apa fualaau'aina, apa fuala'au fai sua, apa supo) In the past month, how often did **you** eat food packaged in a metal can (for example: tinned fish, canned fruit, canned vegetable, canned soup)
____ times per day (taimi i le aso) OR _____ times per week (taimi i le vaiaso)
OR
____ times per month (taimi i le masina) _____ Don't Know (Le iloa)

b. I le masina ua tuana'i atu, e fa'afia ona taumafa **e lou alo** meaai tu'u apa? (f/t: apa eleni, apa fualaauaina, apa fuala'au fai sua, apa supo) In the past month, how often did **your child** eat food packaged in a metal can (for example: tinned fish, canned fruit, canned vegetable, canned soup)
____ times per day (taimi i le aso) OR _____ times per week (taimi i le vaiaso)
OR
____ times per month (taimi i le masina) _____ Don't Know (Le iloa)

1.6. ITEM 6

a. I le masina ua tuana'i atu, e fa'afia **ona e** taumafa i meaai tu'u pepa iila e le manaomia le faavevelaina? (f/t: pepa masi, masi, cereal) In the past month, how often did **you** eat foods that were packaged in plastic film/wrap and were not heated or cooked (e.g., granola bars, crackers, cereal)?
____ times per day (taimi i le aso) OR _____ times per week (taimi i le vaiaso)
OR
____ times per month (taimi i le masina) _____ Don't Know (Le iloa)

b. I le masina ua tuana'i atu, e fa'afia ona taumafa **e lou alo** meaa'i tu'u pepa iila, e le manaomia le faavevelaina? (f/t: pepa masi, masi, cereal) In the past month, how often did **your child** eat foods that were packaged in plastic film/wrap and were not heated or cooked (e.g., granola bars, crackers, cereal)?
____ times per day (taimi i le aso) OR _____ times per week (taimi i le vaiaso)
OR
____ times per month (taimi i le masina) _____ Don't Know (Le iloa)

1.7. ITEM 7

a. I le masina ua tuana'i atu, e fa'afia **ona e** taumafa i meaai e teuina I pepa uga e toe faavevela? (f/t :pai faavevela e faatauina sa'o mai le faleoloa, panikeke falai) In the past month, how often did **you** eat foods that were stored in plastic film/packaging while hot (e.g., heated takeaway pies or fried pancakes)?
____ times per day (taimi i le aso) OR _____ times per week (taimi i le vaiaso)
OR
____ times per month (taimi i le masina) _____ Don't Know (Le iloa)

b. I le masina ua tuana'i atu, e fa'afia ona taumafa **e lou alo** meaai e teuina I pepa uga e toe faavevela? (f/t :pai faavevela e faatauina sa'o mai le faleoloa, panikeke falai) In the past month, how often did **your child** eat foods that were stored in plastic film/packaging while hot (e.g., heated takeaway pies or fried panicakes)?
____ times per day (taimi i le aso) OR _____ times per week (taimi i le vaiaso)
OR
____ times per month (taimi i le masina) _____ Don't Know (Le iloa)

1.8. ITEM 8

a. I le masina ua tuana'i atu, e fa'afia **ona e** taumafa i meaai fa'avevela e ufiufi i pepa ufiufi malama/pepa afifi ai meaai e fa'aaogaina ai le masini faavevela meaai, ogaumu oveni po'o le umukuka? In the past month, how often did you eat foods heated in the microwave or oven or Samoan oven in contact with plastic film/wrap
____ times per day (taimi i le aso) OR _____ times per week (taimi i le vaiaso)
OR
____ times per month (taimi i le masina) _____ Don't Know (Le iloa)

b. I le masina ua tuana'i atu, e fa'afia ona taumafa **e lou alo** meaa'i faavevela e utiufi I pea ufiufi malamalama/pepa ufiufi meaai e toe fa'avevela e fa'aaogaina ai le ogaumu uila, oveni po'o le umukuka? In the past month, how often did **your child** eat foods heated in the microwave or oven or samoan kitchen in contact with plastic film/wrap
____ times per day (taimi i le aso) OR _____ times per week (taimi i le vaiaso)
OR
____ times per month (taimi i le masina) _____ Don't Know (Le iloa)

1.9. ITEM 9

a. I le masina ua tuana'i atu, e fa'afia **ona e** taumafa i meaa'i e fa'avevela i le ogaumu uila e fa'aogaina ai le ipu uga lapoa? In the past month, how often did **you** eat foods that were microwaved in plastic containers or plastic bowl?
____ times per day (taimi i le aso) OR _____ times per week (taimi i le vaiaso)
OR
____ times per month (taimi i le masina) _____ Don't Know (Le iloa)

b. I le masina ua tuana'i atu, e fa'afia ona taumafa **e lou alo** meaa'i e fa'avevela i le ogaumu uila e fa'aogaina ai le ipu uga lapoa? In the past month, how often did **your child** eat foods that were microwaved in plastic containers or plastic bowl?
____ times per day (taimi i le aso) OR _____ times per week (taimi i le vaiaso)
OR
____ times per month (taimi i le masina) _____ Don't Know (Le iloa)

Table S1. BPA-related dietary questions and exposure scores.

Item	Food or Beverage Item	Exposure Scores			
		Literature	Expert 1	Expert 2	Average[a]
Item 1	Cold beverages from a hard plastic cup	1	2	1	1.33
Item 2	Hot beverages from a hard plastic cup	1	3	2	2
Item 3	Canned beverages	3	1	1	1.67
Item 4	Disposable, plastic bottled beverages	1	1	1	1
Item 5	Food packaged in a metal can	3	3	3	3
Item 6	Unheated/uncooked food packaged in plastic/film wrap	0	0	0	0
Item 7	Hot food stored in plastic film/packaging	0	0	0	0
Item 8	Food heated in the microwave/oven in contact with plastic	3	3	2	2.67
Item 9	Food microwaved in plastic containers	2	3	2	2.33

Items correlate to full list of questions presented above; Exposure scores extracted from Tse et al (2017) supplementary material; [a]Average exposure score used in our analyses.

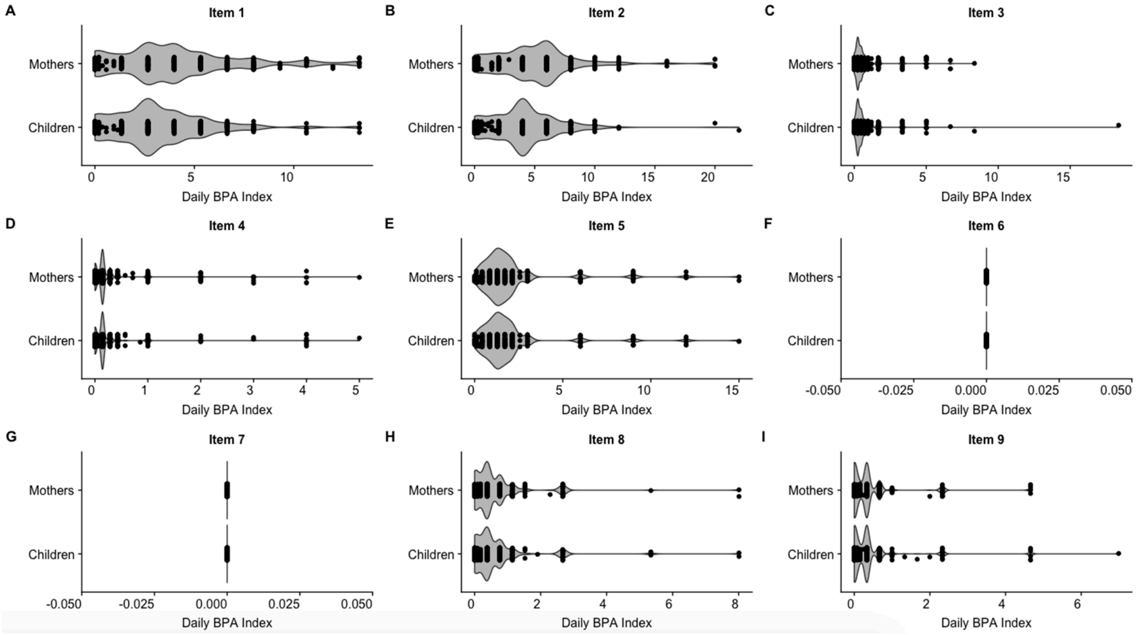

Figure S1. Sina plots for daily, item-specific BPA indices for mothers and children.

Sina plots of daily, item-specific daily BPA indices for mothers in children for (A) Item 1, range of 0 to 13.3 (mothers and children); (B) Item 2, range of 0 to 20 (mothers) and 0 to 22 (children); (C) Item 3, range of 0 to 8.4 (mothers) and 0 to 18.4 (children); (D) Item 4, range of 0 to 5 (mothers and children); (E) Item 5, range of 0 to 14 (mothers and children); (F) Item 6 (BPA exposure score for this item was zero, so the resultant item-specific BPA index is also zero for all participants), (G) Item 7 (BPA exposure score for this item was zero, so the resultant item-specific BPA index is also zero for all participants); (H) Item 8, range 0 to 8.01 (mothers and children); and (I) Item 9, range of 0 to 4.7 (mothers) and 0 to 7.0 (children); See Table S1 for item descriptions.

Table S2. Spearman's rank-order correlations between daily, item-specific BPA indices for mothers and children.

**Item**	**Spearman's rho**[a]
Item 1	0.69
Item 2	0.46
Item 3	0.70
Item 4	0.80
Item 5	0.92
Item 6	NA[b]
Item 7	NA[b]
Item 8	0.86
Item 9	0.84
Total BPA index	0.70

[a]All p-values < 2.2E-16; [b]Item-specific exposure score was 0, thus, consumption frequencies for items 6 and 7 are not BPA-relevant; See Table S1 for item descriptions.

Table S3. Results of multiple linear regression examining associations between categorical daily BPA index and body mass index and abdominal circumference in mothers (n=351).

		**BMI (kg/m2)**			AC (cm)	
	**β**	**95% CI**	*p*	β	95% CI	*p*
Categorical Daily BPA Index[a]						
Low (Tertile 1)			Reference			
Moderate (Tertile 2)	0.45	-1.29 to 2.19	0.61	0.69	-3.03 to 4.41	0.72
High (Tertile 3)	0.18	-1.67 to 2.04	0.85	-1.42	-5.40 to 2.56	0.48
Age (years)	0.07	-0.008 to 0.16	0.08	0.25	0.07 to 0.42	0.01
Physical Activity						
> 0 MVPA minutes		*Reference*			*Reference*	
0 MVPA minutes	-0.79	-2.50 to 0.91	0.36	-0.79	-4.45 to 2.87	0.67
Annual Household Income						
<5,000 talā		*Reference*			*Reference*	
5,000 to 9,999 talā	-0.26	-1.98 to 1.46	0.77	-1.34	-5.03 to 2.35	0.47
≥10,000 talā	3.29	1.23 to 5.34	0.002	5.46	1.05 to 9.86	0.02
Household Assets	0.12	-0.06 to 0.31	0.20	0.21	-0.20 to 0.61	0.31

BPA, Bisphenol A; BMI, Body Mass Index; AC, Abdominal Circumference; β, unstandardized regression coefficient estimate; CI, Confidence Interval; *p*, p-value based on the t-test for the particular adjusted regression coefficient; MVPA, daily moderate to vigorous physical activity minutes; [a]Categorical Daily BPA Index in tertiles of low (Daily BPA Index ≤ 10), intermediate (Daily BPA Index of > 10 to ≤ 13.7), and high (Daily BPA index of > 13.7); R^2 for BMI model, 6.4%; R^2 for AC model, 6.3%; All variance inflation factors < 1.2.

Table S4. Results of Multiple Linear Regression Examining Associations Between Categorical Daily BPA Index and Body Mass Index and Abdominal Circumference in Children (n=370).

	**BMI (kg/m2)[a]**			AC (cm)[b]		
	**β**	**95% CI**	***p***	β	95% CI	*p*
Categorical Daily BPA Index[a]						
Low (Tertile 1)			Reference			
Moderate (Tertile 2)	0.05	-0.44 to 0.53	0.85	-0.01	-1.21 to 1.20	0.99
High (Tertile 3)	0.29	0.22 to 0.80	0.27	0.50	-0.77 to 1.77	0.44
Age (years)	0.20	-0.008 to 0.41	0.06	1.89	1.38 to 2.41	2.03E-12
Sex						
Female		*Reference*			*Reference*	
Male	0.23	-0.17 to 0.63	0.26	0.47	-0.51 to 1.46	0.35
Dietary Pattern						
More modern		*Reference*			*Reference*	
Less modern	-0.05	-0.46 to 0.35	0.79	-0.63	-1.63 to 0.38	0.22
Physical Activity						
More active than peers		*Reference*			*Reference*	
About the same as peers	-0.20	-0.66 to 0.27	0.13	-0.09	-1.24 to 1.06	0.88
Less active than peers	-0.61	-1.38 to 0.17	0.41	-1.94	-3.87 to -0.01	0.05
Annual Household Income						
<5,000 talā		*Reference*			*Reference*	
5,000 to 9,999 talā	-0.48	-1.00 to 0.03	0.07	-0.63	-1.91 to 0.65	0.33
≥10,000 talā	-0.17	-0.78 to 0.44	0.58	0.00	-1.51 to 1.51	1.00
Household Assets (total number)	0.11	0.06 to 0.16	5.23E-05	0.20	0.07 to 0.33	2.10E-03

BPA, Bisphenol A; BMI, Body Mass Index; AC, Abdominal Circumference; β, unstandardized regression coefficient estimate; CI, Confidence Interval; *p*, p-value based on the t-test for the particular adjusted regression coefficient; MVPA, daily moderate to vigorous physical activity minutes; [a]Categorical Daily BPA Index in tertiles of low (Daily BPA Index ≤ 8.63), intermediate (Daily BPA Index of > 8.63 to ≤ 12), and high (Daily BPA index of > 12); R^2 for BMI model, 7.7%; R^2 for AC model, 17.4%; All variance inflation factors < 1.3.
